# Chronic Constipation: Is a Nutritional Approach Reasonable?

**DOI:** 10.3390/nu13103386

**Published:** 2021-09-26

**Authors:** Massimo Bellini, Sara Tonarelli, Federico Barracca, Francesco Rettura, Andrea Pancetti, Linda Ceccarelli, Angelo Ricchiuti, Francesco Costa, Nicola de Bortoli, Santino Marchi, Alessandra Rossi

**Affiliations:** 1 Gastrointestinal Unit, Department of Translational Sciences and New Technologies in Medicine and Surgery, University of Pisa, 56124 Pisa, Italy; massimo.bellini@med.unipi.it (M.B.); barracca.federico@gmail.com (F.B.); rettura.fra@gmail.com (F.R.); pancio10@alice.it (A.P.); l.ceccarelli@ao-pisa.toscana.it (L.C.); a.ricchiuti@int.med.unipi.it (A.R.); fcosta@med.unipi.it (F.C.); nicola.debortoli@unipi.it (N.d.B.); santino.marchi@unipi.it (S.M.); 2 Clinical and Experimental Medicine–Rheumatology Unit, University of Pisa, 56100 Pisa, Italy; alessandra.rossi@unipi.it

**Keywords:** chronic constipation, food, fiber, water, diet, nutrition

## Abstract

Chronic constipation (CC) is one of the most common gastroenterological diagnoses in clinical practice. Treatment includes several steps, depending on the severity of symptoms. Lifestyle modifications and increased intake of fiber and water are suggested by most health professionals. Unfortunately, the recommendations in this regard are the most varied, often conflicting with each other and not always based on solid scientific arguments. This paper aims to clarify this topic by providing practical indications for the management of these patients in every day clinical practice. The literature available on this topic is scarce, and dietary studies have important methodological biases. However, fiber, mainly by binding water and acting as bulking agents and/or as prebiotics for the intestinal microbiota, and mineral water, especially if rich in magnesium and/or bicarbonate, are useful tools. An adequate, well-designed diet should be a cornerstone of any effective treatment for chronic constipation. High-quality studies on larger samples are mandatory to give scientific validity to the role of the food in CC therapy and to enable professionals to choose the best approach for their patients, combining nutritional and pharmacological agents.

## 1. Introduction

Chronic Constipation (CC) is a heterogeneous and extremely troublesome disorder ranked in the top five most common diagnoses among gastroenterologists’ outpatient medical examinations [1]. CC affects about 12% of the world population, with a higher prevalence among females and elderly people [2,3,4].

The most widely used criteria to diagnose CC are the Rome Criteria (Table 1) [5]. 

The Rome criteria, mainly used for scientific purposes, separate functional constipation (FC) from irritable bowel syndrome with constipation (IBS-C). These two different categories should be mutually exclusive. However, in clinical practice, many gastroenterologists have difficulties in clearly separating these two entities, because between them exists a significant overlap, which should be better considered as a continuum [6].

Moreover, constipation can also be due to drug consumption (i.e., opioids) or to other diseases rather than a primary disorder. To further complicate this complex situation, it is important to point out that many patients consider themselves to be constipated without satisfying the Rome criteria. They simply report generic unsatisfying defecation, whose management is not easy because it escapes the traditional diagnostic and therapeutic framework [6].

CC, however, has a negative impact on social and professional life (absenteeism and loss of productivity), reduces the quality of life, and is a heavy economic burden due to the costs related to its diagnosis, treatment, and management [2,7,8].

It is well known that dietary suggestions are the first line treatment for CC, in the opinion of both gastroenterologists and general practitioners (GPs), because CC has always been traditionally considered as being mainly linked to wrong dietary choices [7,9]. Unfortunately, even if many studies are available about CC nutritional management and many different diets have been suggested, mainly by increasing dietary fiber and water intake, there is still little high-quality evidence on the efficacy of dietary interventions [10,11]. The different types of comparator groups used in the various trials, the difficulty of finding a real sham diet for the placebo group, and the different parameters used for evaluating the improvement, are only some of the potential sources of bias that could explain the relatively low quality of evidence. These problems could be solved by well-designed randomized controlled trials (RCTs) using large numbers of patients and similar comparator groups [10].

However, in recent years, the nutritional approach to gastrointestinal diseases has gained a renewed and increasing popularity among both health care professionals and patients. Many people think of food as the main source of their well-being and tend to overestimate the positive effects of some compounds contained in food. This is often purely on the basis of a personal whim, without any expert supervision regarding the diet’s nutritional adequacy and its therapeutic efficacy [12].

The increasing attention paid to the possible effects of food, both positive and negative, on general health has favored the commercialization of a large series of products of varying kinds, including food supplements, functional foods, and nutraceuticals. By *food supplement*, we mean concentrates of nutritional substances (e.g., vitamins, amino acids, salts) in pre-dosed forms, intended to integrate the common diet in cases of chronic or temporary deficiencies. *Functional foods* are those naturally rich or artificially enriched in specific components, which can offer a benefit beyond the common diet. Finally, with the term *Nutraceuticals*, whose name is composed of the words “nutrient” and “pharmaceutical”, we mean those products that are closer to the world of drugs, moving away from food, taking on the aspect of prevention of chronic diseases or used in addition to conventional therapy. These may include products such as fermented red rice-based products for the treatment of hypercholesterolemia, which can be recommended before statin therapy [13].

The aims of the present paper are:

- to analyze the possible impact of water, fiber, and, in general, of the dietary approach on CC;
- to help physicians, dietitians and nutritionists, and, in general, healthcare professionals to understand where they should focus their attention when communicating with patients to provide practical and reliable advice about this topic. This involves both detecting false opinions and misconceptions and suggesting a more correct and healthier nutritional approach on the basis of the scientific evidence available.

## 2. Diet Components in Constipation Relief

### 2.1. Mineral Water

#### 2.1.1. How It Works

The first line treatment for CC involves an adequate intake of water (1.5–2 L/day), even though this is not based on strong scientific evidence. Despite the colon’s high capacity for absorption of ingested water, the laxative action of waters, especially those rich in magnesium and sulfates, has been known for a long time [14]. 

Waters rich in magnesium sulfate owe their laxative effectiveness mainly to the magnesium compounds they contain. Magnesium hydroxide (Mg(OH)_2_) is commonly used as an osmotic laxative at doses greater than 2 g/day. In the stomach, this reacts with the protons (H^+^) of the gastric acid, producing Mg^2+^ and water. However, if taken in high doses, Mg(OH)_2_ is converted in the gut to magnesium carbonate (MgCO_3_), absorbing water from the intestinal walls and hydrating and softening the stool [15,16]. Excessive use of this type of water is not recommended in elderly patients with renal insufficiency because the risk of hypermagnesemia could be higher [16].

The main effect of magnesium is the osmotic effect due to its incomplete absorption. However, other mechanisms are being hypothesized, such as an increased secretion of cholecystokinin (CCK) and Peptide YY (PYY), which in turn modulate intestinal motility, of nitric oxide synthase (NOS), which acts on smooth muscle, and of aquaporin-3 (AQP-3), which regulates the secretion of water in the lumen [17,18]. Moreover, a further mechanism of action could be due to sulfates, which can have a prebiotic action, i.e., they act as a substrate selectively utilized by host microorganisms, thereby conferring a benefit on sulfate-reducing bacteria, as defined by ISAPP in 2016 [15,19].

Bicarbonate-alkaline waters probably act through the serotoninergic system, as suggested by Fornai et al. Indeed, the laxative effect of a bicarbonate alkaline water in mice was antagonized by alosetron (5-HT3 antagonist), even if the increase in colonic reflexes due to the stimulation of duodenal osmoreceptors, together with an action on gastrin and CCK secretion, cannot be ruled out [20,21]. 

#### 2.1.2. Clinical Evidence 

The efficacy of a carbonated calcium/magnesium sulfate-rich mineral water was investigated in 100 FC patients through a double-blind, randomized, placebo-controlled study [22]. The mineral water had a total mineralization content of 2.666 mg/L (573 mg/L calcium, 105 mg/L magnesium, 1.535 mg/L sulfate) and 2.650 mg/L of carbon dioxide. FC patients received 1 L/day mineral water or carbonated tap water (placebo) for 6 weeks. After a 3 week treatment with sulfate-rich mineral water, bowel frequency improved compared with treatment with tap water, but the difference was no longer significant after 6 weeks.

In another controlled trial in FC women, daily consumption of 1 L of magnesium-sulfate mineral water reduced constipation and hard or lumpy stools in a greater percentage of patients than a similar consumption of a natural low-mineral water. This occurred as early as the second week of treatment. The total mineralization content was 2513 mg/L (549 mg/L calcium, 119 mg/L magnesium, 1530 mg/L sulfate, 14.2 mg/L sodium, 4.1 mg/L potassium, 383.7 mg/L bicarbonate, and 4.3 mg/L nitrate) [23]. 

Anti et al. explored the effects of fluid supplementation associated with a high-fiber diet in 177 patients with FC, who were randomly divided into two groups [24]. For two months, both groups consumed a standard diet, providing approximately 25 g fiber per day. Group 1 (58 patients) was allowed water intake ad libitum, while group 2 was instructed to drink 2 L of a bicarbonate-alkaline water per day (113.7 mg/L sodium, 11.6 mg/L potassium, 30.5 mg/L magnesium, 206.1 calcium, 689.3 mg/L bicarbonate). Compliance was monitored throughout the study, and the results were assessed in terms of bowel movement frequency and laxative use. The increased stool frequency in patients taking 25 g daily fiber intake was significantly enhanced by increasing fluid intake to 2.0 L/day. 

See Table 2 for the characteristics of the waters examined by the clinical studies.

### 2.2. Dietary Fiber

#### 2.2.1. How It Works

Guidelines for chronic constipation are generic, recommending that patients “increase fiber intake” [14,25].

Dietary fiber, defined as “the remnant of plant components that are resistant to hydrolysis by human alimentary enzymes” is a class of non-digestible carbohydrates resistant to gastric acids and hydrolysis by digestive enzymes, while “functional” fiber is defined as isolated, non-digestible carbohydrates that have beneficial physiological effects in humans [26,27,28,29]. The fiber group also includes lignin, a highly branched non-polysaccharide polymer, which complies with the definition of dietary fiber, although it cannot be classified as a carbohydrate. Finally, some digestible polysaccharides are still classified as dietary fibers because, when inside the food matrix, they are not reached by the digestive enzymes, such as resistant starch type 1 [30]. 

In the colon, fiber may be fermented by the microbiota, with production of gas (CO_2_, CH_4_, H_2_) and short chain fatty acids (SCFAs), i.e., butyrate, acetate, and propionate, which create osmotic load, accelerating intestinal transit [31,32,33,34]. Moreover, butyrate, which is an important source of energy for the colonic mucosa, also acts at the level of the neurons of the myenteric plexus, increasing gut motility [35,36].

Fiber can retain water, increasing the hydration of the stool. In this context, it is important to point out that the consistency of the stool is closely related to its water content, and even minimal variations can lead to changes in fecal consistency [37]. A normal stool contains 74% of water, whereas a hard stool has less than 72% and a soft stool at least 76%. Therefore, a percentage of as little as 2% in water content can make a difference to stool form [38]. This small variation in consistency allows the stool to be more rapidly moved distally by the peristaltic waves of the colon and more easily evacuated. The peristaltic waves differ according to their amplitude and frequency. The hardest stools are mainly propelled by high amplitude waves, whereas low amplitude waves, which are more frequent during the day, are mainly linked to gas or soft stool movement. Therefore, a small variation in water content also allows better exploitation of the propulsive activity of low amplitude waves, thus resulting in an increase in daily bowel movements (BMs) [38].

The various kinds of fiber are often considered as a homogenous group with the same characteristics, but they are quite different in terms of solubility, fermentability, and viscosity, having different effects at the gastrointestinal level [33]. According to their structure, they can be divided into short- and long-chain carbohydrates. 

The short-chain carbohydrates include fructo-oligosaccharides (FOS) and galacto-oligosaccharides (GOS). These favor the growth of Bifidobacteria and, consequently, the production of large quantities of SCFAs, as well as gas. This can contribute to the onset of the most important side effect of fiber, i.e., bloating [11,39]. 

Fiber-types can be further classified according to their:

- solubility, which depends on their hydrophilicity (physical property of molecules to bind with water), and varies according to the degree of polymerization of the molecule;
- viscosity (degree of resistance to flow);
- fermentability (ability to be metabolized by bacteria in the absence of oxygen) [40].

Solubility also has an impact on fermentability as it increases both the distribution of the molecule along the intestine and its metabolism by gut microbiota.

Fiber can therefore be further divided into:

- Soluble, viscous, fermentable (e.g., Guar gum)
- Soluble, viscous, unfermentable (e.g., Psyllium, HPMC—Hydroxypropyl methylcellulose)
- Soluble, non-viscous, fermentable (e.g., Inulin, FOS, GOS, Pectin)
- Soluble, non-viscous, unfermentable (e.g., PHGG—Partially Hydrolyzed Guar Gum)
- Insoluble and slowly fermentable (e.g., Wheat bran, Resistant starch)
- Insoluble and unfermentable (e.g., Cellulose, Lignin)

Rapidly fermented soluble fiber is found in legumes, wheat, potatoes, rice, barley and rye. It acts as a prebiotic, thereby increasing biomass (and indirectly fecal mass), with consequent production of SCFAs and gas.

Soluble fiber that is only moderately fermented acts by retaining large quantities of water, forming gels, normalizing fecal consistency, and it is the most widely studied fiber as regards its action on FC and IBS-C [38].

Insoluble types of fiber, such as wheat bran, act on intestinal transit by means of an irritative stimulus on the mucosa, which, in turn, induces secretion of water and mucus. However, this is achieved only by the larger and coarse bran particles, while the finer and smooth ones have not been shown to share this laxative property [38,41,42].

#### 2.2.2. Clinical Evidence

A systematic review by Suares et al. analyzed six RCTs in CC patients, four of which used soluble fiber (three used psyllium and the fourth a combination of inulin and maltodextrose) and two used insoluble fibers, such as wheat bran and rye bread [43]. Compared to the baseline, soluble fiber led to an improvement in global symptoms, straining, pain on defecation, and stool consistency. It also increased the mean number of BMs per week and decreased the number of days between BMs. Conversely, data regarding insoluble fiber were conflicting.

Another meta-analysis by Yang et al. showed that dietary fiber had a significant advantage over the placebo only in terms of stool frequency. There was no significant difference in stool consistency, laxative use, and painful defecation between the two groups [44]. 

A systematic review by Rao et al. recently analyzed 11 studies [45]. Due to the study heterogeneity and methodological quality, it was not possible to perform a meta-analysis. However, fiber was beneficial in five out of seven studies involving CC patients and in three out of three involving IBS-C patients. The authors conclude that fiber supplementation is beneficial in mild to moderate CC and IBS-C patients. However, larger, more rigorous, and long-term RCTs are needed.

In conclusion, despite the fact that fiber for CC has relatively little support from large RCTs, it is often suggested as a first-line treatment, and both the European guidelines and the American College of Gastroenterology position paper recommend fiber, mainly the soluble kinds, for CC management [14,46].

### 2.3. The Role of Food 

Food could play a key role in the pathophysiology and treatment of CC. Its beneficial effect is due not only to the fiber content, but also to the presence of other substances (i.e., polyphenols, sorbitol, etc.) synergistically acting and enhancing their beneficial effect. Furthermore, the presence of fiber within food, and not isolated in a pharmaceutical formulation, greatly influences its effect on the bowel, because some intrinsic food features, such as particle size and matrix porosity, can modify the fiber availability for the action of digestive enzymes [30].

For obvious reasons, it is impossible to analyze the mechanisms linked to all kinds of foods, so we only focused on some foods frequently recommended to constipated patients due to their laxative properties.

A randomized clinical trial compared the effects of dried plums and psyllium in patients with CC [47]. Forty subjects were enrolled in an 8 week, single-blind, randomized cross-over study. Subjects received either dried plums (50 g b.d., fiber = 6 g/day) or psyllium (11 g b.d., fiber = 6 g/day) for 3 weeks each, with a 1 week washout period. The number of complete spontaneous BMs per week (primary outcome measure) and stool consistency scores improved significantly with dried plums when compared to psyllium. The authors hypothesize that the most powerful laxative effect of dried plums is due to the presence of sorbitol (14.7 g/100 g) and polyphenols (184 mg/100 g), in addition to the fiber. Indeed, sorbitol acts as an osmotic laxative and holds onto water.

Kiwi fruits are well known for their laxative properties. They are high in vitamin C and contain a wide range of other nutrients, such as fiber, potassium, vitamin E and folate, and various bioactive components. The latter include an array of antioxidants, phytonutrients, and enzymes, all able to provide functional and metabolic benefits [48].

In a study carried out by Rush et al., 42 subjects aged >60 years were recruited [49]. During the 6 week study participants were randomly assigned to one of two trial groups. One group consumed kiwi fruit only in the first 3 week period, while the other group consumed kiwis only for the second 3 week period. Kiwi fruit consumption was set at one kiwi (100 g) per 30 kg of body weight per day. Kiwi consumption was associated with a significant increase in frequency of defecation, stool volume and softness, and ease of defecation. The authors conclude that kiwi fruits were palatable and effective to treat constipation in the elderly. The authors hypothesize a key role for dietary fiber in kiwi fruit, but also the potential role of actinidin. Actinidin is a cysteine protease with proteolytic activity enhancing protein digestion and decreasing gastrointestinal transit time [50]. The mechanism of action of Actinidin is still unclear. A recent hypothesis suggests that Actinidin cleaves the protein kiwellin into kissper and KiTH. Kissper is a small, anionic, cysteine-rich 39-residue peptide acting as an ion channel activator and as a modulator of GI motility. Both Kissper and KiTH display a range of beneficial activities, including an increase in anti-inflammatory response, a reduction in oxidative stress at the GI mucosal interface, and pH dependent and voltage-gated pore-forming activity, together with anion selectivity and channeling [51,52].

Other protease cysteines, apart from actinidine, which are potentially effective on CC, are found in pineapple, papaya, and figs (i.e., bromelain, papain, and ficin) [51].

In animal models, these enzymes prompt an improvement in protein digestion, decrease gastrointestinal transit time, and have an anti-inflammatory action [53,54,55].

Another study carried out by Eady et al. investigated whether daily consumption of three gold-fleshed kiwi fruit could alleviate constipation and improve gastrointestinal discomfort in mildly constipated individuals [56]. Thirty-two participants were enrolled in a 16-week randomized, single-blind, crossover study. Participants received either three kiwis (5 g of fiber/day) or 14.75 g Metamucil ^®^ (5 g dietary fiber/day) for 4 weeks with a 4 week washout between treatments. The number of complete spontaneous BMs per week were significantly greater during daily consumption of three kiwis compared with the baseline and the Metamucil^®^ treatment. Stool consistency also improved with the kiwi fruit producing softer stools and less straining. Gastrointestinal discomfort, abdominal pain, and constipation improved during the kiwi fruit consumption and constipation during the Metamucil^®^ intervention. The authors conclude that daily consumption of three gold-fleshed kiwis is associated with a significant increase in bowel frequency and a reduction in pain and gastrointestinal discomfort.

Chey et al. report a partially randomized, comparative effectiveness trial evaluating kiwifruit, psyllium, and prunes in 79 patients with CC [57]. Eligible patients had <three complete spontaneous bowel movements (CSBMs) per week and were partially randomized to green kiwifruit (2/d), prunes (100 g/d), or psyllium (12 g/d) for 4 weeks. There was a significant increase in weekly CSBM rate with all three treatments; stool consistency significantly improved with kiwifruit and prunes; straining significantly improved with kiwifruit, prunes, and psyllium. Patients randomized to the kiwifruit group reported significant improvement in bloating scores. The authors conclude that kiwifruit, prunes, and psyllium improve constipation symptoms in patients with CC.

Additionally, fig paste, in a study carried out on a constipation rat model, was able to improve constipation, increasing fecal output and water content, and decreasing transit time [58]. The results were confirmed by a randomized, double-blind, placebo-controlled study investigating 80 FC patients [59]. Ficus carica paste supplementation administered for 8 weeks showed a greater improvement in colon transit time, stool consistency, and abdominal discomfort compared with the placebo.

From a mechanistic point of view, the beneficial effects of F. carica paste on constipation are most likely related to its composition. F. carica contains high amounts of cellulose, phenols, flavonoids, and anthocyanins, which are reported to have laxative effects. These bioactive substances may stimulate the chloride channel and/or serotonergic signaling, which, in turn, stimulate colonic secretion of water, electrolytes, and mucin. In addition, fiber in F. carica can also decrease colonic mucinase activity so as to increase the mucin content and enhance the frequency of BMs [59]. 

Flaxseed (Linum usitatissimum) is an oil-based seed containing high amounts of alpha-linolenic acid, linoleic acid, lignans, fiber and many other bioactive components. Nowadays, flaxseed is known as a functional food with many health benefits for humans [60]. In particular, flaxseed could be a safe and effective treatment for constipation because it is a good source of soluble and insoluble fiber. Indeed, 50 g of flaxseed contain 13.3 g of dietary fiber, corresponding to about 50% of the recommended daily intake [61].

Moreover, due to its lipid content and mucilaginous component, flaxseed also has lubricating and stool-softening properties [62]. In a recent randomized controlled trial, 90 patients with FC were enrolled: 60 patients assumed flaxseed flour-enriched meals (50 g/day) and 30 patients lactulose (15 mL/day) for 4 weeks. Patients treated with flaxseed flour reported an improvement in bowel movement frequency and abdominal pain severity and less difficult defecation than those taking lactulose [62]. Furthermore, Hanif Palla et al., studying flaxseed in mice, showed that laxative effects were mediated primarily through a cholinergic pathway with weak histaminergic effects [63].

Some studies suggest that probiotics and fermented milk can improve defecation in constipated patients. However, the mechanism of fermented milk containing probiotics on constipation remains poorly understood. Wang and colleagues recently studied 26 volunteers with chronic constipation symptoms treated with 200 g/d of fermented milk containing Lactobacillus casei Zhang and Bifidobacterium animalis ssp. lactis V9 for 4 weeks [64]. After the intervention a significant improvement of constipation symptoms was observed. The authors speculate that it was due to three potential mechanisms: an improvement of gastrointestinal microbiota, a decrease in inflammation, and a positive action on the regulating metabolic pathways.

Maki et al. evaluated the probiotic effects of kefir-fermented milk for preventing constipation in 42 persons with mental and physical disabilities [65]. The participants were administered 2 g of lyophilized kefir with each meal for 12 weeks. The intake of kefir significantly reduced constipation, compared with the baseline. The authors conclude that adding kefir to the daily diet might benefit persons who have chronic, but not severe, constipation. Kim et al. studied the effect of the ear mushroom (Auricularia) on FC in 34 patients [66]. They conducted a double blind study with 3 groups (placebo, ear mushrooms alone, and ear mushrooms with additives including cascara sagrada, etc.), and found that, in both the ear mushrooms alone and ear mushrooms with additives groups, the frequency of bowel movements, straining, sense of incomplete evacuation, stool consistency, and satisfactory relief all significantly improve.

## 3. Suggestions for Everyday Practice

Simple and correct nutritional advice, which is easy to put into practice, should be given to CC patients in order to avoid inadequate and/or harmful nutritional choices and unnecessary sacrifices. 

It is mandatory to follow a balanced diet, not to skip meals, especially breakfast. Indeed, food intake is able to trigger the gastrocolic reflex, i.e., increasing colonic motility in response to gastric distension, with a synergistic effect with the orthocolic reflex, which takes place at the transition from supine to upright position, causing the propagation of high amplitude colonic peristaltic waves [67]. It is recommended not to ignore the defecatory stimulus. Indeed, the current rhythms of everyday life often force patients to postpone defecation, leading to a progressive decrease in the perception of the defecatory stimulus and, consequently, the need to evacuate.

Adult women should consume approximately 25 g of fiber daily, men approximately 38 g, and children 19–25 g daily. Unfortunately, most people consume only about half this amount of recommended fiber (Table 3).

However, it is worth highlighting that a fiber intake higher than 50 g/day is not necessary and may even be troublesome, causing abdominal distension and flatulence.

The correct fiber intake can be reached by consuming four or five portions of fruit and/or vegetable per day and preferring whole grains, which are rich in fiber and micronutrients.

It is better to eat raw or steamed vegetables than cooked ones, because cooking them (e.g., in the oven or a pot) can alter some beneficial substances (e.g., vitamins, polyphenols), even if the dietary fiber is not destroyed. 

A useful suggestion could be to prepare just one course, combining vegetables with the main meal in order to improve flavor and guarantee the adequate daily intake. 

Among cooked vegetables, it is preferable to choose spinach, green beans, aubergines, and fennel to be consumed boiled or steamed. Artichokes, broccoli, and cauliflower are also recommended, even if they can induce abdominal bloating. Vegetable soups are an alternative way to take the recommended vegetable portions. These dishes can be seasoned using extra virgin olive oil and seeds, such as flaxseed.

Kiwis, pears, apples, apricots, dried plums, and figs are the most frequently recommended fruits for CC management. They can be eaten both during and outside of meals. Bran and powdered fiber supplements may be helpful when a sufficient amount of fiber is not taken through food. They can be added to cereals, yogurt, fruit juices, or soups. The enrichment of the diet with fiber should be slow and gradual in order to avoid (or reduce) disturbances such as bloating, flatulence, and intestinal cramps.

The consumption of at least eight glasses (1.5–2 L) of fluids daily is recommended to facilitate the laxative effectiveness of fiber intake. 

It is worth recalling that gastric obstruction and fecal impaction may occur when fiber is consumed without a sufficient fluid intake. This is mainly the case in patients with neuromuscular disorders, dysmotility syndromes, chronic opioid use, and pelvic floor disorders, in whom an increased dietary fiber intake should be carefully evaluated [67].

It is important that this advice is given in a clear and comprehensive way to the patient, that is carrying out appropriate food education. This can also be done thanks to the help of new means of communication such as social networks or dedicated apps.

An active lifestyle is also recommended because the evidence seems to indicate that physical inactivity is an important etiological factor of CC [68].

Table 4 provides a short summary of general recommendations to give CC patients.

Particular attention has to be paid to IBS patients who are often offered a low-FODMAP (Fermentable oligo-, di-, monosaccharides, and polyols) diet, which has been shown to be effective in improving the painful abdominal symptoms, and bloating [11,69]. At least in its first phase, this approach may reduce fiber intake resulting in a reduction in short-chain soluble carbohydrates. These draw water into the gut lumen and are fermented by the intestinal microbiota, producing gas and SCFAs. A reduction in FODMAPs could therefore reduce the transit of osmotic fluid into the intestinal lumen, increasing the risk of constipation. For the prevention of this constipating effect, it is necessary to have the intervention of an expert nutritionist who is able to find low-FODMAP alternatives, especially in IBS patients with constipation.

## 4. Conclusions

The nutritional approach has an important role in CC treatment in everyday clinical practice, because it is the cornerstone on which CC therapy should be built [7,9]. Hence, it should no longer be considered simply as an “old wives’ tale”. 

Unfortunately, this approach meets many obstacles on its path. Indeed, there is still a shortage of high-quality studies available. Moreover, there are various obstacles to drawing reliable conclusions, which should be based on reliable systematic reviews or metanalyses. These often include the lack of adequate sample size, the different kinds of comparators used in the different trials, the difficulty of finding a real placebo, and the different parameters used for evaluating the efficacy of fiber, water, and food in treating CC [10,11].

Another important point to note is that the cause of CC is not the same for all patients. This obviously also affects the response to different therapies. For example, a slow transit constipation is more likely to respond to a change in the diet, in which the increase in fiber and the use of mineral water can increase the hydration of the stool and therefore speed up intestinal transit. That is, provided that the patient does not exceed with the supplementation of fibers which could worsen bloating and stool consistency. On the contrary, a dyssynergic defecation has more difficulty in responding to dietary changes only. In these patients, due to the pelvic floor dyssynergia, the correct therapy is pelvic floor rehabilitation. The role of the dietary changes in this case is more blurred, but can help to improve fecal consistency, preventing the production of hard stools, which would be more difficult to expel [12].

Another important issue is that clinical trials on a nutritional approach to CC are mainly carried out on patients collected within a gastroenterological setting. Gastroenterologists generally treat CC patients with more severe symptoms, who have probably already experienced dietary modifications without satisfactory results. Indeed, patients with milder symptoms, who could be more likely to improve their condition by using only a nutritional approach, are not often enrolled in studies carried out in gastroenterological centers.

Furthermore, the impact of a great deal of fake news about the positive or negative effects of nutrition available on internet and social networks should be clearly addressed. Fake news is sometimes more convincing for public opinion than the suggestions provided by health professionals. Therefore, there is still much work to do in order to provide the public with reliable information about food and the correct use of the different kinds of mineral waters and fiber. There are significant differences in terms of symptom improvement and possible drawbacks related to CC.

It is necessary that physicians have more in-depth knowledge about the therapeutic role of an adequate nutritional regimen. It is therefore advisable to insist on the training of medical personnel given that there is a wide range of different approaches commonly used today by GPs and gastroenterologists [9,71]. Unfortunately, thorough nutritional education is not routinely given during undergraduate courses and residency. This could be one of the causes leading to a poor perception of the need for a multidisciplinary approach to CC, which should involve GPs, gastroenterologists, and nutrition professionals.

## Figures and Tables

**Table 1:** Diagnostic Criteria for Functional Constipation ^a^.

1. Must include 2 or more of the following:
(a) Straining during more than ¼ (25%) of defecations
(b) Lumpy or hard stools (Bristol Stool Scale 1–2) more than ¼ (25%) of defecations
(c) Sensation of incomplete evacuation more than ¼ (25%) of defecations
(d) Sensation of anorectal obstruction/blockage more than ¼ (25%) of defecations
(e) Manual maneuvers to facilitate more than ¼ (25%) of defecations
(f) Fewer than 3 spontaneous bowel movements per week
2. Loose stools are rarely present without the use of laxatives
3. Insufficient criteria for irritable bowel syndrome

^a^ Criteria fulfilled for the last 3 months with symptom onset at least 6 months prior to diagnosis.

**Table 2:** Description of the specific waters studied for treatment of constipation, their chemical composition, doses used, and outcomes.

Water	Chemical Composition	Doses	Outcomes
Calcium/magnesium sulfate-rich mineral water	573 mg/L calcium, 105 mg/L magnesium, 1.535 mg/L sulfate, and other 2.650 mg/L carbon dioxide	1 L/day	Bowel frequency improved
Magnesium-sulfate mineral water	549 mg/L calcium, 119 mg/L magnesium, 1.530 mg/L sulfate, 14.2 mg/L sodium, 4.1 mg/L potassium, 383.7 mg/L bicarbonate, 4.3 mg/L nitrate	1 L/day	Bowel frequency improved
Bicarbonate-alkaline water	113.7 mg/L sodium, 11.6 mg/L potassium, 30.5 mg/L magnesium, 206.1 calcium, 689.3 mg/L bicarbonate.	2 L/day	Bowel frequency improved

**Table 3:** Selected food sources of fiber [70].

Food	Grams Per Serving	% Daily Value
Almonds^—28 g	3.3	13
Apple^—1 medium	3.3	13
Artichoke *—1 piece	6.5	26
Banana^—1 medium	3.1	12
Black beans **—½ cup	7.5	30
Bran ready-to-eat cereal—½ cup	8.8	35
Broccoli *—½ cup	2.8	11
Chickpeas *—½ cup	6.2	24
Figs, dried, ¼ cup	3.7	14.5
Green peas *—½ cup	4.4	18
Lentils *—½ cup	7.8	31
Navy beans **—½ cup	9.5	38
Oat bran—¼ cup	3.6	14
Orange^—1 medium	3.1	12
Peas *—½ cup	2.5	10
Prunes^—½ cup	3.8	15
White beans **—½ cup	6.3	25

* cooked, ** canned, ^ raw Daily Values (DVs) are the recommended amounts of nutrients to consume each day. The %DV is how much a nutrient in a single serving of an individual packaged food contributes to your daily diet.

**Table 4:** Dietary and behavioral recommendations for CC patients.

1. Consume an adequate amount of fiber in the diet, in the form of whole grains, vegetables, fruits, and legumes
2. Drink at least eight glasses of water (1.5–2 L) a day
3. Follow a balanced diet with an adequate intake of macro- and micronutrients
4. Have an active lifestyle
5. Do not ignore the defecatory stimulus
